# Small volume of hypertonic saline as the initial fluid replacement in experimental hypodynamic sepsis

**DOI:** 10.1186/cc4901

**Published:** 2006-04-13

**Authors:** Alejandra del Pilar Gallardo Garrido, Ruy Jorge Cruz, Luiz Francisco Poli de Figueiredo, Maurício Rocha e Silva

**Affiliations:** 1 Research Division, Heart Institute (InCor), University of Sao Paulo School of Medicine, Sao Paulo, Brazil

## Abstract

**Introduction:**

We conducted the present study to examine the effects of hypertonic saline solution (7.5%) on cardiovascular function and splanchnic perfusion in experimental sepsis.

**Methods:**

Anesthetized and mechanically ventilated mongrel dogs received an intravenous infusion of live *Escherichia coli *over 30 minutes. After 30 minutes, they were randomized to receive lactated Ringer's solution 32 ml/kg (LR; *n *= 7) over 30 minutes or 7.5% hypertonic saline solution 4 ml/kg (HS; *n *= 8) over 5 minutes. They were observed without additional interventions for 120 minutes. Cardiac output (CO), mean arterial pressure (MAP), portal and renal blood flow (PBF and RBF, respectively), gastric partial pressure of CO_2 _(pCO_2_; gas tonometry), blood gases and lactate levels were assessed.

**Results:**

*E. coli *infusion promoted significant reductions in CO, MAP, PBF and RBF (approximately 45%, 12%, 45% and 25%, respectively) accompanied by an increase in lactate levels and systemic and mesenteric oxygen extraction (sO_2_ER and mO_2_ER). Widening of venous-arterial (approximately 15 mmHg), portal-arterial (approximately 18 mmHg) and gastric mucosal-arterial (approximately 55 mmHg) pCO_2 _gradients were also observed. LR and HS infusion transiently improved systemic and regional blood flow. However, HS infusion was associated with a significant and sustained reduction of systemic (18 ± 2.6 versus 38 ± 5.9%) and mesenteric oxygen extraction (18.5 ± 1.9 versus 36.5 ± 5.4%), without worsening other perfusional markers.

**Conclusion:**

A large volume of LR or a small volume of HS promoted similar transient hemodynamic benefits in this sepsis model. However, a single bolus of HS did promote sustained reduction of systemic and mesenteric oxygen extraction, suggesting that hypertonic saline solution could be used as a salutary intervention during fluid resuscitation in septic patients.

## Introduction

Widespread microcirculatory abnormalities and cellular alterations leading to an uncoupling between blood flow and metabolic tissue requirements, particularly in splanchnic perfusion, are common in septic patients and have been implicated in sepsis-related mortality due to multiple organ dysfunction [1-3]. While adequate oxygen supply to the gastrointestinal tract is the key factor for maintaining physiological intestinal function, it is well known that there is a discrepancy between systemic and regional variables, and that interventions that increase systemic oxygen delivery do not necessarily result in improved regional perfusion [4-8]. Therefore, therapeutic interventions that prevent or rapidly reduce the severity of intestinal mucosal hypoperfusion may improve outcome in septic patients.

Although early fluid resuscitation has been shown to improve cardiovascular function and outcome in experimental and human sepsis [9-11], the optimal fluid therapy is a matter of controversy. In spite of adequate systemic hemodynamic restoration, hypoperfusion of the intestinal mucosa may persist and result in deranged barrier function [4-8,12-14]. In a previous study, we found that an early, large volume of crystalloid after live *Escherichia coli *injection in dogs promoted partial and transient benefits, essentially during the fluid infusion, which were especially poor at the splanchnic bed [5].

Hypertonic saline (HS), with or without colloidal solution, has been successfully used for treating hemorrhagic shock in animal and clinical studies [12,13,15]. Immediate plasma volume expansion due to intracellular fluid mobilization, particularly from erythrocytes and endothelial cells, promotes systemic hemodynamic benefits and microcirculatory blood flow improvement [12,13,15-18]. Moreover, in comparison with lactated Ringer's solution (LR) resuscitation, we demonstrated that HS resuscitation reduces bacterial translocation and lung injury after hemorrhage [19], and avoids morphological alterations in bone marrow seen after hemorrhagic shock [20]. It has also been shown that hypertonic saline reduces neutrophil-endothelium interactions and vascular leakage [21], and attenuates neutrophils' cytotoxic response post-injury [22]. Both hemodynamic and immunomodulatory effects of hypertonic resuscitation may provide a potential benefit for the treatment of sepsis [15,16,18-22].

The experience with HS solutions in sepsis is limited. Therefore, we performed the present study to test the hypothesis that HS infusion promotes superior systemic and regional benefits than conventional isotonic crystalloid infusion in experimental sepsis.

## Materials and methods

This study was approved by the Animal Care and Use Committee of the University of São Paulo Medical School and conducted in compliance with the guidelines of the National Regulations for the Care and Use of Laboratory Animals.

### Animal preparation

Fifteen healthy male mongrel dogs weighing 14 to 20 kg each were fasted for 12 hours before the study, with free access to water. Anesthesia was induced with an intravenous injection of 0.1 mg/kg of morphine sulfate followed by 25 mg/kg of sodium pentobarbital. Additional doses of pentobarbital (2 mg/kg) were used whenever required. A cuffed endotracheal tube was placed in the trachea to allow mechanical ventilation with a 1.0 inspired fraction of oxygen, at a tidal volume of 15 ml/kg (670 Takaoka ventilator, São Paulo, Brazil). Respiratory rate was adjusted to maintain arterial partial pressure of CO_2 _(pCO_2_) at 40 ± 5 mmHg. A urinary bladder catheter was placed for urinary drainage. During surgical preparation, a heating pad was used to maintain normothermia, and the animals received LR 20 ml/kg/h to compensate for fluid losses. Each animal received an intravenous injection of 1.5 mg/kg of ranitidine.

The right common femoral artery was cannulated with a polyethylene catheter (PE240) to measure mean arterial pressure at the abdominal aorta and to collect arterial blood samples for blood gas and lactate analysis. Through the right common femoral vein, a catheter (PE240) was introduced for fluid infusion.

A balloon-tipped catheter (5-Fr ARROW^® ^Balloon Thermodilution Catheter Inc., Pennsylvania, PA, USA) was inserted into the pulmonary artery through the right external jugular vein under guidance of pressure waves, as displayed by a multichannel monitor system. This catheter was connected to a cardiac computer (Vigilance™, Baxter Edwards Critical Care, Irvine, CA, USA) to measure cardiac output, using 3 ml bolus injections of isotonic saline at 20°C. All catheters were connected to disposable pressure transducers (Transpac Disposable Transducer, Abbott, Chicago, IL, USA) and to a computerized multichannel system for biological data acquisition (Acknowledge^® ^III MP 100 WSW, Biopac Systems Inc., Goleta, CA, USA).

Through a midline laparotomy, an ultrasonic flow probe (Transonic Systems Inc., Ithaca, NY, USA) was placed around the portal vein for transit time flow measurement (T206 Transonic Volume Flowmeter, Transonic Systems). A P240 catheter was threaded into the portal system via inferior mesenteric vein for portal blood sampling. The abdominal cavity was then carefully closed. After the surgical preparation was completed, the animals were allowed to recover for 30 minutes before the measuring protocol was initiated and the infusion of LR was discontinued.

A large gastric polyethylene tube was orally introduced and placed in the stomach to allow gastric lavage and drainage with warm isotonic saline solution until a clear fluid was obtained. A tonometry catheter (16 F TRIP™, Datex-Ohmeda Division, Instrumentation Corp., Helsinki, Finland) was introduced orally and positioned at the large curvature of the stomach. The tonometry catheter was then connected to a calibrated gas capnometer (Tonocap, model TC-200, Tonometrics, Datex-Ohmeda Division) for gastric mucosal pCO_2 _(PgCO_2_) measurement every 10 minutes.

### Bacterial preparation

A strain of *E. coli *O55, provided by the Department of Bacteriology of Adolfo Lutz Institute, São Paulo, Brazil, originating from the stool of a patient with gastrointestinal sepsis, was used in this study. In brief, according to previous studies [5,7], the bacteria were stored in conservative milieu at room temperature, activated in trypticase soy broth, plated in trypticase soy agar and incubated at 37°C for 24 hours. Aliquots were then suspended in sterile saline. The bacterial suspension was estimated turbidimetrically by comparing the newly grown bacterial suspension to known standards through spectrophotometry at 625 nm to obtain a culture of desired bacterial density. The same suspension was subsequently quantified by plating successive 10-fold dilutions onto trypticase soy agar plates and scoring visible colonies after 24 hours of incubation at 37°C. The target dose, as calculated by the methods outlined above, was 3 × 10^9 ^cells/ml or 0.6 × 10^10 ^cfu/ml. Then, 1.2 × 10^10 ^cfu/kg of body weight was used to induce sepsis.

### Data collection and analysis

Mean systemic and pulmonary arterial pressures, heart rate, and portal vein blood flow were continuously recorded. Cardiac output was determined using the thermodilution technique and expressed as cardiac index, according to the estimated body surface area. Each determination was the arithmetic mean of three consecutive measurements when their differences did not exceed 10%. Central venous blood temperature was recorded from the thermistor in the pulmonary artery catheter.

Blood gases, hemoglobin, hematocrit, sodium and blood lactate levels were obtained from arterial, portal and mixed venous samples at baseline (T0), then at 30, 60, 90, 120, 150, 180 and 210 minutes after the initiation of the bacterial infusion. All blood samples were analyzed by a Stat Profile Ultra Analyzer (Nova Biomedical, Waltham, MA, USA). The arterial oxygen content (CaO_2_), mixed venous oxygen content (CvO_2_), portal oxygen content (CpO_2_), systemic oxygen delivery (sDO_2_), mesenteric oxygen delivery (mDO_2_), systemic oxygen extraction ratio (sO_2_ER), mesenteric oxygen extraction ratio (mO_2_ER), systemic oxygen consumption (sVO_2_), mesenteric oxygen consumption (mVO_2_) and arteriovenous oxygen content difference (C(a-v)O_2_) were calculated using standard formulae.

The following pCO_2 _gradients were calculated: D(v-a)pCO_2_, as the difference between mixed venous pCO_2 _and arterial pCO_2_; D(p-a)pCO_2_, as the difference between portal vein pCO_2 _and arterial pCO_2_; D(g-a)pCO_2_, as the difference between gastric mucosal pCO_2_, measured by gas tonometry, and arterial pCO_2_.

### Experimental protocol

**Figure 1:**
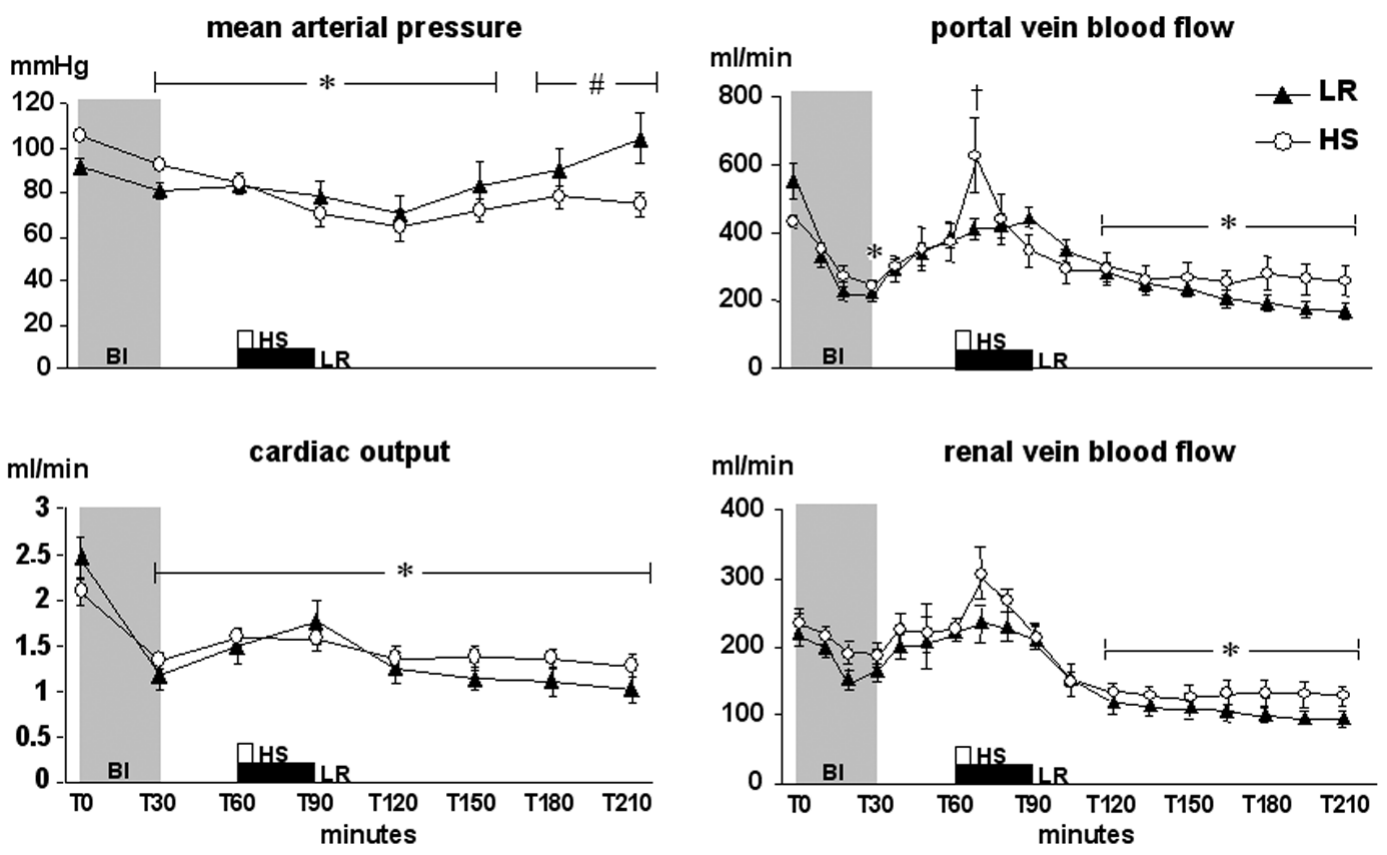
Mean arterial pressure (mmHg, mean ± standard error of the mean (SEM)), cardiac output (l/minute, mean ± SEM), portal vein blood flow (ml/minute, mean ± SEM) and renal vein blood flow (ml/minute, mean ± SEM) during the experimental protocol. LR group received lactated Ringer's solution 32 ml/kg over 30 minutes, *n *= 7; HS group received 7.5% hypertonic saline solution 4 ml/kg over 5 minutes, *n *= 8. *p < 0.05 versus T0, both groups; ^†^*p* < 0.05 versus HS; ^# ^HS, *p *< 0.05 versus T0. BI, bacterial infusion.

After stabilization, baseline (T0) measurements were obtained (Figure 1). The infusion of *E. coli*, in a dose of 1.2 × 10^10 ^cfu/kg, was initiated and maintained for 30 minutes in all groups (T30). After 30 minutes of observation (T60), the animals were randomized into two groups: fluid treatment with LR 32 ml/kg (*n *= 7) over 30 minutes or 7.5% HS 4 ml/kg (*n *= 8) over 5 minutes. The animals were observed without additional interventions for 120 minutes (T210). They were then euthanized by a pentobarbital overdose followed by hypertonic potassium chloride injection.

### Statistical methodology

Results are presented as mean ± standard error of mean (SEM). Statistical analysis was performed using a Statistic Package for Social Sciences for Windows software (version 6.0, SPSS Inc., Chicago, IL, USA). Differences between groups were analyzed using repeated measure analysis of variance and *post hoc *Tukey's test. Linear correlation was tested using the Spearman rank method. Statistical significance was considered for *p *values less than 0.05.

## Results

### Effects of live *E. coli *infusion

**Figure 2:**
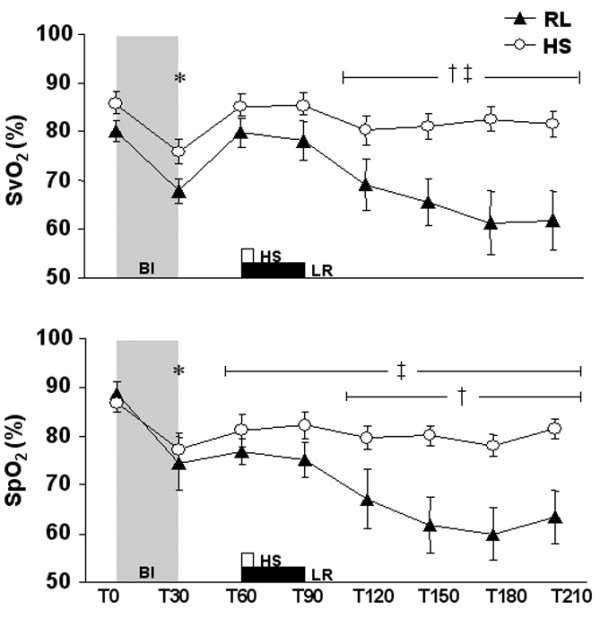
Mixed venous (SvO_2_) and portal (SpO_2_) oxygen saturation (%, mean ± standard error of the mean) during the experimental protocol. LR group received lactated Ringer's solution 32 ml/kg over 30 minutes, *n *= 7; HS group received 7.5% hypertonic saline solution 4 ml/kg over 5 minutes, *n *= 8. *p < 0.05 versus T0, both groups; ^†^*p *< 0.05 versus HS; ^‡^LR, *p *< 0.05 versus T0. BI, bacterial infusion.

**Figure 3:**
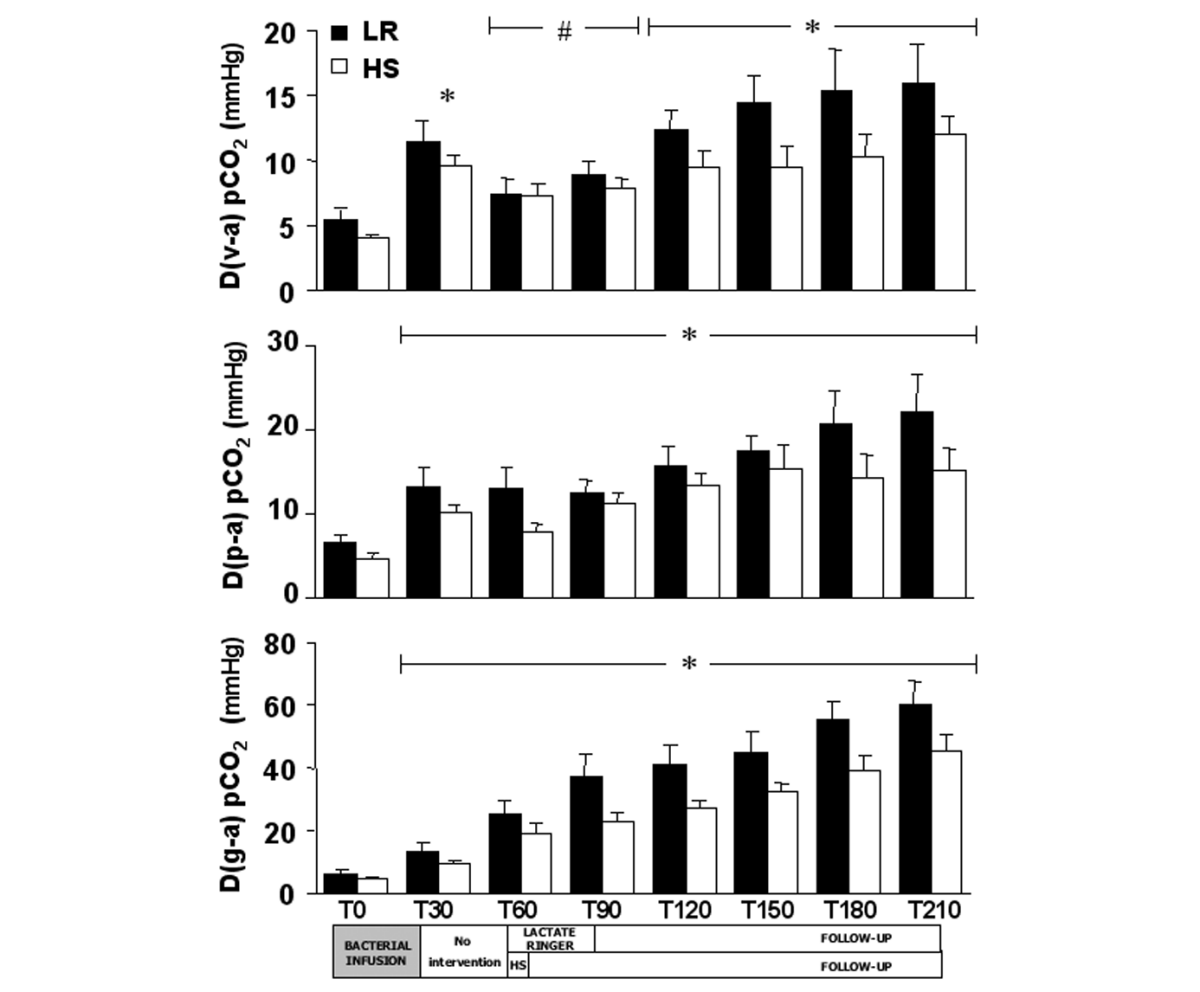
Venous-arterial pCO_2 _gradient (D(v-a)pCO_2_; mmHg, mean ± standard error of the mean (SEM)), portal-arterial pCO_2 _gradient (D(p-a)pCO_2_; mmHg, mean ± SEM) and gastric mucosal-arterial pCO_2 _gradient (D(g-a)pCO_2_; mmHg, mean ± SEM) during the experimental protocol. LR group received lactated Ringer's solution 32 ml/kg over 30 minutes, *n *= 7; HS group received 7.5% hypertonic saline solution 4 ml/kg over 5 minutes, *n *= 8. *p < 0.05 versus T0, both groups; ^# ^HS, *p *< 0.05 versus T0.

Live *E. coli *infusion resulted in a hypodynamic sepsis state. In spite of minor changes in mean arterial pressure and renal blood flow, an immediate and severe impairment of cardiac output and portal blood flow were observed (Figure 1). These hemodynamic changes were accompanied by blood gas changes indicative of widespread metabolic deterioration, including progressive increases in systemic and mesenteric oxygen extraction rates (Tables 1 and 2) with a decrease in mixed venous and portal oxygen saturations (Figure 2), raised arterial and portal lactate levels (Tables 1 and 2), metabolic acidosis (Table 1), and widening of pCO_2 _gradients (Figure 3). No significant changes in systemic or regional oxygen consumption were detected throughout the experiment (Tables 1 and 2). Hemoglobin levels increased in both groups after bacterial infusion (Table 1).

### Effects of fluid replacement

Fluid infusion with a large volume of LR or a small volume of HS promoted similar hemodynamic effects. Both fluid replacement regimens were associated with transient increases in cardiac output (Figure 1) and portal and renal blood flow (Figure 1), while increased systemic and mesenteric oxygen extraction were ameliorated (Tables 1 and 2). Fluid infusion did not change the progressive increases of pCO_2 _gradients (D(v-a)pCO_2_, D(p-a)pCO_2 _and D(g-a)pCO_2_) in both groups (Figure 3). By the end of the experimental protocol, no significant differences between the groups could be detected for systemic or regional hemodynamic variables (Figure 1), except for an HS-induced significant and sustained improvement in systemic and mesenteric oxygen extraction (Tables 1 and 2) and, thus, a significant increase of mixed venous and portal oxygen saturation (Figure 2). In spite of fluid infusion, arterial and portal lactate levels showed a similar progressive increase in both groups (Tables 1 and 2). A brief decrease toward baseline hemoglobin levels was observed immediately after fluid infusion in both groups (Table 1). Hypertonic saline infusion induced a sustained increase in serum sodium levels (Table 1).

## Discussion

Using an intravenous injection of live *E. coli*, we reproduced the hemodynamic and metabolic derangement observed in non-resuscitated hypodynamic sepsis, characterized by immediate and marked reduction of systemic and splanchnic blood flow. A large volume of LR or a small volume of HS promoted similar transient hemodynamic benefits, which were unable to restore sepsis-induced perfusional deficits. However, a single bolus of HS did promote sustained systemic and mesenteric oxygen extraction reductions without deterioration of perfusional markers, such as lactate levels and pCO_2 _gradients.

Experimental data have shown a sepsis-induced impairment of tissue oxygen extraction that contributes to an imbalance between DO_2 _and VO_2_, possibly due to microcirculatory dysfunction [23] and cytopathic hypoxia [24]; thus, reductions in oxygen extraction would be deleterious in septic settings. However, Rivers and colleagues [9] have found that early goal-directed therapy guided by restoration of central venous saturation, which decreases sO_2_ER in septic patients, was associated with significant benefits related to outcome when it was applied at an earlier stage of disease. In this context, since we did not detect additional worsening in any systemic or regional tissue oxygen marker, we can assume that the reduction in sO_2_ER and mO_2_ER, in this model, is a salutary effect of HS.

In stable septic patients, the use of HS/hydroxyethyl-starch [25] and HS/dextran solution [16] produced transient increases in cardiac output and DO_2_, similar to our findings. However, they found no changes in oxygen extraction, which could be explained by a previous hemodynamic resuscitation with adequate volume and catecholamine infusion. Thus, either there was no significant oxygen debt due to the already elevated oxygen delivery levels at baseline or the global oxygen measurements used were not able to detect regional hypoxia [16]. In our model, HS and LR were the first fluid replacements after septic insult, resembling the study by Rivers and colleagues [9]. In spite of different amounts of volume infused, the higher SvO_2 _and SpO_2_, as well as a trend toward lower pCO_2 _gradients in HS animals, may be considered a superiority of HS over LR resuscitation. We speculate that HS reduced the oxygen extraction in this model through its capacity to improve microcirculatory blood flow by capillary reopening, redistribution of regional blood flow and ameliorating the mismatch between DO_2 _and VO_2_. However, we could not exclude a possible metabolic effect of HS decreasing oxygen demand as demonstrated experimentally [26].

The sustained high levels of lactate and pCO_2 _gradients after HS infusion could have resulted from an obvious incomplete hemodynamic resuscitation. Moreover, high lactate levels could be explained by mechanisms other than anaerobic metabolism [5,16]. In this scenario, the widening of pCO_2 _gradients could have resulted from decreased blood flow with an increase in tissue transit time and decreases in pulmonary blood flow, or from an increase in aerobic production of CO_2 _without the required proportional increase of blood flow [5,12,13,27].

Animal and clinical studies suggest that the microcirculatory alterations are independent of systemic hemodynamic changes. The perfusion of gut villi was markedly decreased in septic rodents compared to hypovolemic controls with a similar degree of hypotension [28]. Norepinephrine infusion in septic patients increased the mean arterial pressure and cardiac output, whereas skin microvascular blood flow and gastric mucosal pCO_2 _remained unchanged [29]. In accordance with the above studies, comparing our septic model with controlled hemorrhagic shock in dogs [13], we observed that higher mean arterial pressure, cardiac output, and systemic and regional oxygen delivery in septic animals resulted, paradoxically, in higher D(g-a)pCO_2 _gradients than those observed in animals subjected to hemorrhagic shock [13]. Additionally, while in septic animals the increase in D(g-a)pCO_2 _was three-fold higher than the increase observed in the D(p-a)pCO_2 _gradient, in hemorrhagic animals it was only two-fold higher. These phenomena reflect the more profound effect of hypoperfusion on the gut mucosal layer versus the mesenteric bed as a whole, and highlight the worst derangement of sepsis-induced gut perfusion, probably due to the association of altered blood flow, microvascular dysfunction and tissue metabolic disturbances. We also observed that the D(p-a)pCO_2 _gradient accompanied the changes of portal blood flow while the D(g-a)pCO_2 _gradient did not parallel either systemic or regional blood flow trends, suggesting that microcirculatory blood flow distribution within the gastrointestinal wall could not be predicted from macrocirculatory regional or systemic blood flow measurements. In fact, we and others have failed to demonstrate a good correlation between the gastric mucosal-arterial pCO_2 _gradient and hepatosplanchnic blood flow [5,12-14,30].

Oi and colleagues [17] showed in a porcine endotoxin shock model that a 7.5% saline/6% dextran-70 infusion improved cardiac output, portal and intestinal mucosal blood flow and ameliorated the intestinal-arterial pCO_2 _gradient compared to isotonic (0.9%) saline/6% dextran-70. These benefits were also accompanied by lower mortality [17]. Several reasons could explained these divergent findings. First, we did not associate dextran, a colloid whose antithrombotic, hemorheological and blood cell-endothelial cell interaction modulating effects are well known [17]. Secondly, HS in the presence of colloid provides plasma volume expansion for a longer period of time [15]. Moreover, while we evaluated the same sodium load in different volumes, they used the same volume with different sodium loads.

Both sepsis and fluid infusion may induce endothelial and red blood cell edema, which reduces the capillary lumen, and increases viscosity and hydraulic resistance, leading to an early compromise of microcirculatory blood flow. The large crystalloid volume required during initial resuscitation in septic patients, about 6 to 10 liters, in the first hours [2,9], results in hemodilution of plasma proteins and reduction of colloid osmotic pressure. It leads to interstitial fluid accumulation, which may worsen gas exchange, decrease myocardial compliance, limits oxygen diffusion to the tissues, and contributes to metabolic acidosis [2,31]. Moreover, a large crystalloid volume can itself result in fluid overload, where the lung and gut are primarily affected, and is associated with primary and secondary intra-abdominal hypertension, decreased intestinal perfusion, prolonged mechanical ventilation, increased incidence of multiple organ failure and death [32-34]. It should be noted that we obtained similar hemodynamic benefits with a small volume of HS (almost eight times smaller than in resuscitation using LR), which could ultimately avoid the aforementioned deleterious effects of fluid overload.

Our animals were not fully resuscitated with both fluids regimens, and this could be a possible explanation for the maintenance of a comparable D(g-a)pCO_2 _in both HS and LR groups throughout the experimental protocol. Furthermore, the increase in hemoglobin levels after bacterial challenge and transient decrease after fluid replacement suggests increased microvascular permeability with extravascular redistribution of the infused fluid and sustained hypovolemia. Splenocontraction during septic insult could also contribute to the observed changes in hemoglobin levels during the experimental protocol. However, in a previous study, we observed the same behavior of hemoglobin levels during the bacterial infusion and fluid resuscitation in splenectomized dogs [5].

There are some limitations in our experimental model and protocol. Our model induced an immediate hemodynamic collapse. This behavior is seldom observed in clinical sepsis, although it may mimic some extreme conditions such as meningococcemia, pneumococcal bacteremia in asplenic individuals and severe infections in the presence of profound granulocytopenia [11]. Resuscitation tends to be an ongoing process, while our study followed a strict protocol of fluid replacement, with no additional interventions. The short observation time makes it impossible to analyze the later impact of the fluid resuscitation regimens on the development of multiple organ dysfunction and survival. However, our goal was to address the acute impact of small volume resuscitation on systemic and regional levels, thereby allowing us to detect the long-lasting effects and safety of this intervention.

## Conclusion

We conclude that the early large volume of LR and single small bolus of HS promoted similar hemodynamic benefits in this hypodynamic septic model. Nevertheless, the sustained reductions in systemic and mesenteric oxygen extraction without worsening other perfusional markers in HS animals suggest that it may be a potential tool in the resuscitation of septic patients. Further investigations are required, including longer follow-up periods to appraise the real impact of this intervention on the development of multiple organ dysfunction and survival.

## Key messages

• 	A small volume of HS 7.5% promoted similar hemodynamic and metabolic benefits when compared to a large volume of LR in this sepsis model.

• 	A single bolus of HS as first fluid resuscitation was able to restore the mixed venous and portal oxygen saturation when applied early in the hypodynamic sepsis model.

## Abbreviations

D(g-a)pCO_2 _= difference between gastric mucosal pCO_2 _and arterial pCO_2_; D(p-a)pCO_2 _= difference between portal vein pCO_2 _and arterial pCO_2_; D(v-a)pCO_2 _= difference between mixed venous pCO_2 _and arterial pCO_2_; HS = hypertonic saline; LR = lactated Ringer's solution; mO_2_ER = mesenteric oxygen extraction ratio; pCO_2 _= partial pressure of CO_2_; SEM = standard error of mean; sO_2_ER = systemic oxygen extraction ratio.

## Competing interests

The authors declare that they have no competing interests.

## Authors' contributions

APGG conceived of the study, ran the experimental protocol, collected and drafted the manuscript. RJCJ ran the experimental protocol, helped the statistical analysis and drafted the manuscript. LFPF performed the statistical analysis and helped to draft the manuscript. MRS participated in experimental design and helped to draft the manuscript. All authors read and approved the final version of this manuscript.

## Figures and Tables

**Table 1:** Electrolytic and systemic oxygen-derived variables in both groups

	Bacterial infusion		Fluid resuscitation			Follow up		
	T0	T30	T60	T90	T120	T150	T180	T210

sDO_2 _(ml/minute)								
LR	454 ± 44	214 ± 25^a^	310 ± 37^a^	340 ± 48^a^	246 ± 37^a^	233 ± 32^a^	237 ± 40^a^	222 ± 34^a^
HS	415 ± 28	274 ± 19^a^	350 ± 25^a^	308 ± 38^a^	279 ± 24^a^	292 ± 21^a^	298 ± 13^a^	290 ± 23^a^
sVO_2 _(ml/minute)								
LR	95 ± 11	67 ± 9	64 ± 10	73 ± 10	60 ± 7	68 ± 7	74 ± 7	68 ± 6
HS	77 ± 11	91 ± 14	61 ± 7	52 ± 7	56 ± 6	60 ± 6	55 ± 6	58 ± 6
sO_2_ER (%)								
LR	20 ± 2.1	31 ± 2.3^a^	20 ± 3.7	22 ± 3.8	30 ± 5.1	34 ± 4.7^a,b^	38 ± 6.5^a,b^	38 ± 5.9^a,b^
HS	14 ± 1.9	33 ± 8.3^a^	15 ± 1.8	14 ± 1.7	19 ± 3.2^a^	18 ± 2.5	17 ± 2.3	18 ± 2.6
AL (mmol/l)								
LR	0.65 ± 0.2	1.30 ± 0.3^a^	2.28 ± 0.4^a^	3.57 ± 0.5^a^	3.48 ± 0.6^a^	4.08 ± 0.8^a^	4.60 ± 1.1^a^	4.90 ± 1.3^a^
HS	1.16 ± 0.2	1.76 ± 0.2^a^	2.67 ± 0.3^a^	3.55 ± 0.2^a^	4.02 ± 0.2^a^	4.11 ± 0.2^a^	4.10 ± 0.2^a^	4.20 ± 0.2^a^
HCO_3 _(mmol/l)								
LR	23.0 ± 0.6	22.6 ± 0.8	21.1 ± 0.7^a^	21.1 ± 0.7^a^	20.0 ± 0.5^a^	18.7 ± 0.6^a^	17.6 ± 1.0^a^	17.4 ± 1.2^a^
HS	22.5 ± 0.6	21.8 ± 0.7	20.7 ± 0.5^a^	18.3 ± 0.7	18.6 ± 0.7^a^	18.3 ± 0.7^a^	18.1 ± 0.5^a^	17.6 ± 0.5^a^
Na (mmol/l)								
LR	146.6 ± 0.1	146.7 ± 0.3	147.0 ± 0.5	146.7 ± 0.4^b^	147.6 ± 0.4^b^	146.7 ± 0.7^b^	145.3 ± 0.9^b^	146.1 ± 0.8^b^
HS	145.6 ± 0.7	145.9 ± 0.4	146.4 ± 0.7	157.1 ± 0.7^a^	155.8 ± 0.7^a^	156.5 ± 0.6^a^	155.9 ± 0.7^a^	155.9 ± 0.7^a^
Hb (g/dl)								
LR	12.9 ± 0.4	13.5 ± 0.5	15.1 ± 0.6^a^	13.6 ± 0.3	14.1 ± 0.3^a^	14.4 ± 0.5^a^	15.3 ± 0.4^a^	15.6 ± 0.3^a^
HS	13.6 ± 0.4	14.2 ± 0.4	15.5 ± 0.5^a^	13.3 ± 0.6	14.8 ± 0.7	15.5 ± 0.7^a^	15.9 ± 0.7^a^	16.5 ± 0.7^a^

Bacterial infusion: intravenous infusion of 1.2 × 10^10 ^cfu/kg of live *Escherichia coli *in 30 minutes in both groups. Fluid resuscitation: LR group received lactated Ringer's solution 32 ml/kg over 30 minutes, *n *= 7; HS group received 7.5% hypertonic saline solution 4 ml/kg over 5 minutes, *n *= 8. Follow up: 120 minutes without additional interventions. sDO_2_, systemic oxygen delivery; sVO_2_, systemic oxygen consumption; sO_2_ER, systemic oxygen extraction rate; Na, plasmatic sodium; AL, arterial lactate; HCO_3_, bicarbonate; Hb, hemoglobin. ^a^*p* < 0.05 versus T0, both groups; ^b^*p* < 0.05, LR versus HS.

**Table 2:** Regional metabolic and oxygen-derived variables in both groups

	Bacterial infusion		Fluid resuscitation			Follow up		
	T0	T30	T60	T90	T120	T150	T180	T210

mDO_2 _(ml/minute)								
LR	103 ± 11	42 ± 4^a^	83 ± 10	86 ± 12	57 ± 8^a^	50 ± 6^a^	43 ± 6^a^	38 ± 5^a^
HS	86 ± 3	50 ± 3^a^	85 ± 13	69 ± 10	62 ± 9^a^	60 ± 9^a^	63 ± 9^a^	60 ± 8^a^
mVO_2 _(ml/minute)								
LR	15.0 ± 2.3	11.3 ± 1.9	20.4 ± 2.5	19.7 ± 2.6	13.3 ± 2.7	15.9 ± 2.1	15.3 ± 2.1	10.5 ± 1.5
HS	14.8 ± 2.4	11.6 ± 1.5	13.1 ± 1.7	10.9 ± 1.6	11.5 ± 1.4	11.2 ± 1.2	12.6 ± 1.76	12.1 ± 2.1
mO_2_ER (%)								
LR	11.2 ± 2.5	25.3 ± 5.1	22.8 ± 2.4^a^	24.7 ± 3.4^a^	32.64 ± 6.0^a^	38.0 ± 5.8^a,b^	39.9 ± 5.3^a,b^	36.5 ± 5.4^a,b^
HS	13.2 ± 1.7	22.8 ± 3.4^a^	18.8 ± 3.4	17.7 ± 2.7	20.3 ± 2.3^a^	19.8 ± 2.0	21.9 ± 2.2	18.5 ± 1.9
PgCO_2 _(mmHg)								
LR	42.1 ± 1.7	51.4 ± 3.8^a^	60.2 ± 5.3^a^	72.2 ± 8.1^a^	75.2 ± 7.3^a^	77.0 ± 1.8^a^	84.8 ± 7.1^a^	89.7 ± 7.5^a^
HS	39.8 ± 1.6	45.1 ± 2.0^a^	53.5 ± 3.2^a^	56.0 ± 3.3^a^	62.8 ± 2.9^a^	68.8 ± 3.1^a^	75.5 ± 4.0^a^	80.6 ± 4.1^a^
PL (mmol/l)								
LR	0.97 ± 0.3	1.73 ± 0.4^a^	2.48 ± 0.4^a^	3.70 ± 0.5^a^	3.65 ± 0.4^a^	4.14 ± 0.6^a^	5.21 ± 1.0^a^	4.70 ± 0.9^a^
HS	1.42 ± 0.2	1.98 ± 0.3^a^	2.81 ± 0.31^a^	4.24 ± 0.2^a^	4.88 ± 0.3^a^	4.52 ± 0.2^a^	4.61 ± 0.2^a^	4.52 ± 0.2^a^

Bacterial infusion: intravenous infusion of 1.2 × 10^10 ^cfu/kg of live *Escherichia coli *in 30 minutes in both groups. Fluid resuscitation: LR group received lactated Ringer's solution 32 ml/kg over 30 minutes, *n *= 7; HS group received 7.5% hypertonic saline solution 4 ml/kg over 5 minutes, *n *= 8. Follow up: 120 minutes without additional interventions. mDO_2_, mesenteric oxygen delivery; mVO_2_, mesenteric oxygen consumption; mO_2_ER, mesenteric oxygen extraction rate; PgCO_2_, gastric mucosal pCO_2_; PL, portal vein lactate. Data are expressed as mean ± standard error. ^a^*p *< 0.05 versus T0, both groups; ^b^*p *< 0.05, LR versus HS.
